# The Youth Educating Peers (YEP) project: making adaptions for youth peer-led sexual health education

**DOI:** 10.1177/17579139241303647

**Published:** 2025-07-05

**Authors:** K McCausland, L Geraghty, R Lobo

**Affiliations:** WA Sexual Health and Blood-borne Virus Applied Research and Evaluation Network (SiREN), School of Population Health, Curtin University, Perth, WA, Australia; Collaboration for Evidence, Research and Impact in Public Health, School of Population Health, Curtin University, Perth, WA, Australia; Youth Affairs Council of Western Australia (YACWA), PO Box 334, Leederville, Perth, WA 6903, Australia; WA Sexual Health and Blood-borne Virus Applied Research and Evaluation Network (SiREN), School of Population Health, Curtin University, Perth, WA, Australia; Collaboration for Evidence, Research and Impact in Public Health, School of Population Health, Curtin University, Perth, WA, Australia

## Introduction

The high incidence of sexually transmissible infections (STIs) among Australian young people^1^ and the growing use of digital technologies in young people’s sex lives have underscored the need for integrating digital literacy and online safety education into relationships and sexuality education.^2^ Young people want youth-friendly sexual health and blood-borne virus (SHBBV) education provided by their friends and peers (peer education). Youth centres and youth health services are preferred sources of SHBBV education outside of school and can play a significant role in supporting young people with SHBBV education.^3^

## The Youth Educating Peers Project

The Youth Educating Peers (YEP) project (hereafter ‘the Project’) is coordinated by the Youth Affairs Council of Western Australia (YACWA) and aims to build capacity at the individual, organisation and youth sector^i^ levels to implement peer-based youth sexual health promotion. Initiatives are responsive and timely and reflect the often rapidly evolving contexts of young people. Innovative practices prioritise the integration of research evidence with the expertise of place-based practitioners and the lived experience of young people.

The Project employs a group of paid peer educators aged 18–25 years (the YEP Crew) to give young people opportunities to connect with other young people (12–25 years), access education about the issues that affect their sexual health and take action to improve relationships and SHBBV outcomes. Monitoring and evaluation of the Project includes post-workshop debriefing and evaluation forms; annual youth and youth sector surveys;^4^ staff supervision and mentoring; youth reference group (16–25 years); professional reference group (professionals from the youth and sexual health sectors); and regular academic review of evaluation tools.

**Figure:**
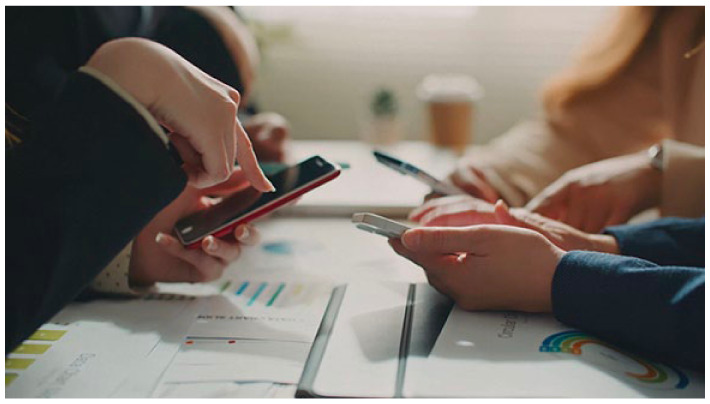


Over the last decade, the Project has undergone substantial adaption, responding to changes within the youth community and SHBBV sector, and the COVID-19 pandemic. Before the pandemic, engagement with the youth sector and young people was largely conducted face-to-face through training for youth workers^ii^ with limited engagement conducted through social media or the YEP website (https://theyepproject.org.au/). The focus was to increase young people’s engagement with sexual health services and increase STI testing.

## How did YEP Adapt during the Covid-19 Pandemic?

During the peak of the COVID-19 pandemic (March to August 2020), the Project’s engagement with the youth sector and young people pivoted to online delivery. Dynamic multimodal responsive relationships and SHBBV education content (e.g. webinars, resources, blogs, videos, and social media initiatives) were created, updated, and delivered by the YEP Crew via the Project’s website and social media platforms – TikTok, Instagram, YouTube, and Facebook. The focus of the Project changed to providing responsive, engaging and diverse SHBBV messaging for young people and the youth sector including sexual health for everyone, safer sex, contraception, anatomy and pleasure, consent and healthy relationships, and sex and technology.

## What Outcomes Resulted from the Change to Online Service Delivery?

Table 1 summarises the outcomes and indicators of the Project after pivoting to online delivery.

**Table 1:** Project outcomes resulting from the change to online delivery.

Outcome	Indicator
The Project documented an increase in engagement with young people and youth workers in regional and remote areas.	• Comments and likes on social media posts included statements from professionals and young people in regional WA about where they lived and their community, these people often then chose to follow the relevant social media account and share the content on their account. • The 2020 YEP Youth Survey included 25% of participants from regional WA, compared to 7% of 2019 respondents. • The 2020 YEP Youth Sector Survey included 42% of regional participants (note: regional residents account for 19% of the WA population). • The YEP Project commenced delivering professional development sessions online in 2020. Each webinar in 2020 included 39%–42% regional participation.
The Project documented an increase in engagement (i.e. views, comments, likes, and shares) from target groups via online platforms.	• Comments and likes on social media posts included statements from professionals and young people in regional WA about where they lived and their community. • SHBBV target population members tagged peers in targeted posts and shared content. • Positive comments from community members gained traction on the posts. • The algorithm on TikTok supported the targeting of population groups and geographical areas. • The 2020 YEP Youth Survey (n = 139) was completed by: ○ 22% Aboriginal and/or Torres Strait Islander young people; ○ 35% migrant, refugee and/or culturally and linguistically diverse young people; ○ 35% trans or gender-diverse young people; ○ 30% young people with a disability. • The 2019 YEP Youth Survey (n = 114) was completed by: ○ 23% migrant, refugee and/or culturally and linguistically diverse young people; ○ 23% trans or gender-diverse young people; ○ 21% young people with a disability; ○ 0% identified as an Aboriginal and/or Torres Strait Islander young person. • 82% of participants of the 2020 Youth Sector Survey were satisfied with the information the YEP Project provided through its online platforms.
The Project documented a significant increase in social media following.	• The YEP Project Facebook page: followers increased to 5011 (2020) from 2828 (2019). • The YEP Project Facebook page: likes increased to 4641 (2020) from 2597 (2019). • The YEP Project Facebook page: reach increased to 668,021 (2020) from 180,717 (2019). • The YEP Project Instagram page: followers increased to 1121 (2020) from 528 (2019). • The YEP Project TikTok account (created in 2020): followers increased to over 1500 (2023) from 882 (end of 2020). • The YEP Project TikTok account (created in 2020): likes increased to 17,400 (2023) from 9682 (end of 2020). • The YEP Project TikTok account (created in 2020): views increased to 250,000 (2023) from 94,974 (end of 2020).
The Project documented an increase in the number of professional development requests.	• The YEP Project was invited to share its strategies for working with young people during government-mandated COVID-19 restrictions to youth and SHBBV professional networks, symposiums and conferences. • The YEP Project exceeded its capacity to deliver professional development sessions due to the high number of requests for online and face-to-face sessions.
The Project documented an increase in knowledge about the Project among young people and the youth sector.	• 70% of participants of the 2020 YEP Youth Sector Survey reported online (website, social media, email, webinars), paper-based (reports, documents, resources) or face-to-face (workshops, stalls, presentations) interaction with the YEP Project, compared with 50% of 2019 respondents. • Participants of the 2020 YEP Youth Survey were asked how they learned about the YEP Project. Responses included 46% Facebook, 10% Instagram, 9% TikTok and 6% YEP Project website. Compared with 2019 results: 11% Facebook, 4% Instagram and 1% YEP Project website.
The Project documented an increase in SHBBV knowledge among young people and the youth sector.	• 77% of participants of the 2020 YEP Youth Survey reported that as a result of viewing YEP Project content online or meeting YEP Project staff face-to-face, their SHBBV knowledge had increased. • 72% of participants of the 2020 Youth Sector Survey believed their knowledge, skills and confidence regarding youth sexual health issues had increased as a result of engaging with the YEP Project in 2020.
The Project documented increased confidence in discussing referral pathways for STI testing.	• 55% of participants of the 2020 YEP Youth Sector Survey reported they were more confident discussing youth sexual health referral pathways with the young people they work with, including access to STI screening, after engaging with the YEP Project. • 67% of participants of the 2020 YEP Youth Sector Survey reported YEP Project resources assisted their collaborative practice, compared with 48% in 2019.

## What was Learnt from the Change to Online Service Delivery?

Key learnings were as follows:

- Consult with young people and the youth sector about relevant SHBBV education topics and messages.
- Provide responsive, accurate, and engaging educational material promptly.
- Provide consistent messages across several channels through a variety of media.
- Collaborate with external agencies that can promote sexual health information.
- Undertake regular engagement with young people and youth workers.
- Create accessible and downloadable materials for people with low literacy and disability.
- Promote messages that are validating and sex-positive and include peers who openly identify with SHBBV target populations.
- Seek and implement feedback from young people.
- Interrogate the success of resources and strategies, and leverage those that work.
- Create ‘trending’ content to increase social media reach (e.g. use trending sounds and create collaborative posts).

## How does YEP Currently Operate?

The current aims of the Project are to educate, empower, and positively evolve young people’s perceptions, attitudes, and behaviours around SHBBV issues through:

1. Youth education: Youth peer-led sexual health workshops, outreach sessions, and engaging online educational content.
2. Youth sector capacity building: Professional development and consultation services.

Consultation by the Project with young people revealed a hybrid model of face-to-face and online engagement was preferred ‘post COVID’. Peer education continues to feature as a youth participation and health promotion strategy and the diverse group (i.e. age, gender, sexuality) of peer educators (i.e. YEP Crew) create high-quality and engaging content.

## Advice for Others Seeking to Implement a Sexual Health Youth Peer Education Project

### Partnership

Youth services should partner with an SHBBV service. The youth service’s role is to support peer educators in delivering sexual health education. The SHBBV service should provide ongoing support through physical and human resources, intellectual property, and clinical guidance.

### Service delivery

Aim to offer multimodal content – online, where most appropriate and accessible, along with face-to-face delivery, for example:

- use a variety of media (e.g. online, print) to acknowledge young people’s varied learning styles and preferred contemporary visual representations of information (e.g. infographics, stories, reels, and posts);
- balance ‘ever-green’^iii^ and ‘trend-specific’ content that is high-quality, engaging, and reflective of young people’s educational needs and interests;
- be accessible to youth workers to increase their SHBBV knowledge and enable content sharing;
- be peer-led or at a minimum co-designed with young people and be guided by clinical evidence and evidence-based practice from the youth and SHBBV sectors; and
- be accessible for people with a variety of disabilities, be trauma-informed and sex-positive, and include representation of people from SHBBV target populations (e.g. sexuality, gender, and ethnically diverse young people).

### Peer educators

Peer educator recruitment activities should be in partnership with relevant community services, key community leaders, and influencers to access people from SHBBV target populations.^5,6^ Peer educators should be provided with ongoing support, supervision, and training, for instance; SHBBV knowledge; trauma-informed practice; administrative, technological, and facilitation skills; strategies to effectively implement boundaries, create inclusive environments, manage challenging behaviours, respond to disclosures, and effectively challenge ableism, sexism, homophobia, and racism). They should also be given guidance on how to be continuously responsive in what and how content is delivered; and be financially reimbursed for their time, including project delivery, training, debriefing, supervision, and organisational and community networking and engagement.^5,6^

### Evaluation

Adaption of existing evaluation tools or development of new tools that are responsive to the dynamic context of youth work is important to ensure appropriate data are collected to demonstrate impact and outcomes. Monitoring and evaluation data can establish programme-level progress and results; inform management and decision-making processes; support accountability; and guide organisational learning for programme improvement.^7^

## Conclusion

The YEP Project provides peer-based education and responsive SHBBV education for young people and was able to successfully pivot to online delivery during the peak of the COVID-19 pandemic. Lessons learned emphasise the importance of consultative approaches, peer-led sexual health education, accurate and consistent information dissemination through a variety of channels, collaboration with external agencies, and the creation of accessible, inclusive, and sex-positive content. Building the capacity of youth workers and peer educators to deliver youth SHBBV education using different modes is important to enable service responsiveness, continuity, and safety of young people.

## Notes

i. The youth sector is a collective name for services and programmes that meet the needs of young people, typically aged 12–25 years. While the majority of youth services are based in the community sector in WA, youth services and youth workers are based in a variety of agencies, including but not limited to faith services, educational services, justice, health, and government agencies serving young people.

ii. Youth work is a practice that places young people and their interests first. Youth work is a relational practice, where the youth worker operates alongside the young person in their context. Youth work is an empowering practice that advocates for and facilitates a young person’s independence, participation in society, connectedness, and realisation of their rights.

iii. Evergreen content is relevant for a long time and provides value to your audience long after you publish it.
